# Drinking-Water

**Published:** 1896-10

**Authors:** 


### EDITORIAL.

The office of the Business Manager of THE JOURNAL is Nos. 311 and 312 Fitten Building.

The editors are not responsible for views expressed by contributors.

Articles for publication should reach this office not later than the 15th inst.

Address all communications, and make all remittances payable to THE ATLANTA MEDICAL AND SURGICAL JOURNAL, Atlanta, Ga.

Reprints of articles will be furnished, when desired, at a nominal cost. Requests for the same should always be made *on the manuscript*.

### TRI-STATE MEDICAL SOCIETY OF ALABAMA, GEORGIA, AND TENNESSEE.

The eighth annual meeting of this society to be held in Chattanooga, Tenn., on October 13, 14, and 15, promises to be both interesting and instructive. A number of papers are already on the list, and a large attendance is hoped for. Dr. J. B. Murfree, Murfreesboro, Tenn., is president, and Dr. Frank Trester Smith, Chattanooga, is secretary.

### DRINKING-WATER.

Our remarks upon the subject of drinking-water in last month's issue may properly be followed by a few words in connection with its domestic purification. We do not intend to discuss the separation of inorganic substances so much as the prevention of access to the body of living germs capable of setting up disease, though processes having the latter in view frequently are more or less successful with regard to the former also. Filtration is the popular device for removing all the possible impurities of water, and as a matter of fact it may be stated that, as usually employed, filtration is more likely to do harm than good. It does harm because the filters are either inefficient, or if at one time efficient, become clogged and add more to the fluid than they remove. No filter whatsoever can be left to take care of itself, and in the frequent absence of proper care, it engenders merely a false feeling of security.

Animal charcoal yields phosphate of lime to the water, a medium suitable to the cultivation of germs, which it does not prevent from passing through its substance. Other filters are made of spongy iron, a mixture of iron, charcoal, and clay, of magnetic carbide of iron of polarite, etc., all of which are better than charcoal, though none of these is quite trustworthy, and all require care and renewal. Some of these act chemically as well as mechanically. The Pasteur-Chamberland filter can be trusted for a time to remove all suspended matter, including germs, so much so, that it is employed in laboratories in bacteriological investigations. But even it will, after a time, permit the passage of bacteria, and it is necessary at intervals to subject it to a high temperature to destroy the organic matter, which gradually gains access to its interior. This filter is composed of a form of Sevres porcelain baked at a great heat, and the water to be filtered is slowly forced through it at a high pressure and by a special arrangement. As quoted in this Journal for August, "Pasteur" filters were recently used during an epidemic of typhoid fever in Scotland, with the apparent effect of completely protecting those using the water prepared in this way. The conclusion arrived at a few years ago by the River Pollution Commissioners (England) was that all the methods of purification by filtration, whether carried out by water companies on the large scale, or by the consumer on his own premises, are inadequate to prevent the propagation of epidemic disease by water. An epidemic of typhoid fever at Lausanne, Switzerland, was caused by the use of water which had passed through the earth for half a mile after contamination, by which filtration fine particles of flour were removed, but not the living germs.

Distillation and boiling are the only sure methods of rendering a polluted water harmless. One soon becomes accustomed to the somewhat insipid taste of boiled water, or it can easily be aerated; in any case the actual security it brings against disease is well worth the slight discomfort; and this is true of milk, which inadvertently, through the washing of the cans, or intentionally, is apt to be mixed with water. Milk loses nothing in the process and gains in digestibility.

